# Effectiveness of mobile application on changing weight, healthy eating habits, and quality of life in children and adolescents with obesity: a randomized controlled trial

**DOI:** 10.1186/s12887-021-02980-x

**Published:** 2021-11-10

**Authors:** Narueporn Likhitweerawong, Nonglak Boonchooduang, Kulnipa Kittisakmontri, Weerasak Chonchaiya, Orawan Louthrenoo

**Affiliations:** 1 Division of Growth and Development, Department of Pediatrics, Faculty of Medicine, Chiang Mai University, 110 Inthawarorot Road, Sriphum, Muang, Chiang Mai, 50200 Thailand; 2 Division of Pediatric Nutrition, Department of Pediatrics, Faculty of Medicine, Chiang Mai University, 110 Inthawarorot Road, Sriphum, Muang, Chiang Mai, 50200 Thailand; 3 Maximizing Thai Children’s Developmental Potential Research Unit, Division of Growth and Development, Department of Pediatrics, Faculty of Medicine, Chulalongkorn University, 1873 Rama 4 Road, Pathumwan, Bangkok, 10330 Thailand

**Keywords:** Childhood obesity, Mobile application, Weight reduction, Eating behavior, Quality of life, Children, Adolescent

## Abstract

**Background:**

A technology-based intervention, such as a mobile application, was a growing interest and potentially effective modality for treating obesity. The study aimed to evaluate the effectiveness of the OBEST, a new mobile/tablet-based application in reducing weight and encouraging healthy eating behaviors and quality of life in children with obesity. The application could assist healthcare professionals to treat children and adolescents with obesity.

**Methods:**

A randomized controlled trial was conducted in the hospital and school settings in Chiang Mai, Thailand. Seventy-seven children and adolescents with obesity were randomized into two groups; one received standard care combined with the OBEST application, and the other received only standard care. The outcomes were changes in weight, healthy eating behaviors, and quality of life assessed by the Pediatric Quality of Life Inventory (PedsQL) from baseline to six-month follow-up between the two groups.

**Results:**

The results showed that the intervention group reduced more body mass index (BMI) and had a higher number of participants engaging in healthy eating behaviors than the standard care group but did not reach a statistically significant level, except for less frequent consumption of fast food. The participants in the intervention group had 4.5 times higher odds of decreased engaging in fast-food consumption than the standard care group at 6 months follow-up (odds ratio, 4.5 [95% CI, 1.41 to 14.35]). There were no statistically significant changes in PedsQL scores over 6 months in between groups.

**Conclusions:**

The current study was unable to detect a significant effect of the OBEST application as an adjunct tool to the standard treatment on reducing weight in obese children and adolescents. However, the mobile application might help to increase engaging in healthy eating behaviors. Further studies with a larger sample are needed to confirm our findings.

**Trial registration:**

The trial was retrospectively registered at the Thai Clinical Trials Registry (trial registration number: TCTR20200604008, on June 4, 2020).

**Supplementary Information:**

The online version contains supplementary material available at 10.1186/s12887-021-02980-x.

## Background

Childhood obesity is a major concern worldwide. The worldwide prevalence of obesity in children and adolescents aged 5-19 has noticeably increased in roughly 40 years. It was found that increased prevalence during 1975 to 2016 was from 0.9 to 7.8% among boys and from 0.7 to 5.6% in girls, respectively [1, 2]. The combination of poor nutrition, excess calorie intake, and lack of physical activity have contributed to weight gain [3]. Apart from the biological and genetic factors, the socio-cultural, economic, and physical environmental factors strongly put people at risk of being obese [4]. Many obese children grow into obese adolescents and obesity in adult life unless they are appropriately treated [5]. Serious consequences such as diabetes, cardiovascular diseases, and other non-communicable diseases have arisen at younger ages in children and adolescents with obesity [5]. Obesity does not only affect physical health but many children and adolescents with obesity also suffer from psychological problems and have a poor quality of life. Many mental illnesses including depression, anxiety, substance use, and eating disorders, are also considered unfavorable outcomes of obesity [6]. Wallander et al. reported that children with obesity had a lower health-related quality of life than non-overweight/non-obese children, particularly in psychosocial functioning [7].

Therapeutic interventions for childhood obesity include nutritional therapy, increasing physical activity, reducing sedentary behaviors, psychological intervention, medication, and surgery [8]. A multimodal approach involving lifestyle interventions such as dietary changes, physical activity, and behavioral interventions was recommended as a mainstay of treatment and essential for long-term maintenance of weight loss [9, 10]. However, it was difficult to successfully treat children and adolescents with obesity, as seen in a systematic review presenting the small effect of non-pharmacological weight-loss treatment [11]. The evidence of not achieving behavior changes has also been reported in previous studies [12–14]. Failure of weight reduction and remaining unhealthy behaviors could be from a lack of motivation and poor adherence to treatment [15, 16]. Therefore, it is necessary to find new adjunctive tools to build motivation and improve adherence among those obese patients.

Technology-based modalities are aimed to enhance the efficacy of obesity treatment, such as web-based, interactive, and smartphone application interventions. To date, a mobile application has been a growing interest and promising tool in treating obesity [17]. Although several mobile application studies focused on weight reduction, most developed mobile applications did not show a significant decrease in weight and body mass index (BMI) [18]. While some previous studies proved the mobile applications could enhance lifestyle changes, like eating behaviors in children and adolescents with obesity [19–21], the results reflecting the effectiveness of mobile applications in improving healthy eating habits were mixed and inconclusive.

This study aimed to evaluate the OBEST application’s effectiveness in reducing weight (primary outcome) and improving eating habits and quality of life (secondary outcomes) in obese children and adolescents. We hypothesized that the children and adolescents with obesity would lose weight, change to healthier eating habits, and improve their quality of life after receiving the application added to the standard care at a six-month follow-up.

## Methods

### Study design

This study was a two-arm parallel design randomized controlled trial evaluating the effectiveness of the OBEST application in reducing weight and improving healthy eating behaviors and quality of life in children and adolescents with obesity. The study was conducted between May 2018 and December 2019. The participants were randomized into the group receiving the intervention (OBEST application added to the standard care) and receiving standard care in a 1:1 ratio using a block size of four randomization from a web-based randomization system. The random allocation sequence was provided by a research assistant. The allocation concealment was managed by using opaque envelopes. The outcome assessors were blinded to the treatment allocation. Ethical approval for this study was obtained from the Research Ethics Committee of the Faculty of Medicine at Chiang Mai University (180/2561). The study protocol was registered with the Thai Clinical Trials Registry (trial registration number: TCTR20200604008). Written informed consent/informed assent was obtained from all parents and participants in the study.

### Participants

**Fig. 1:**
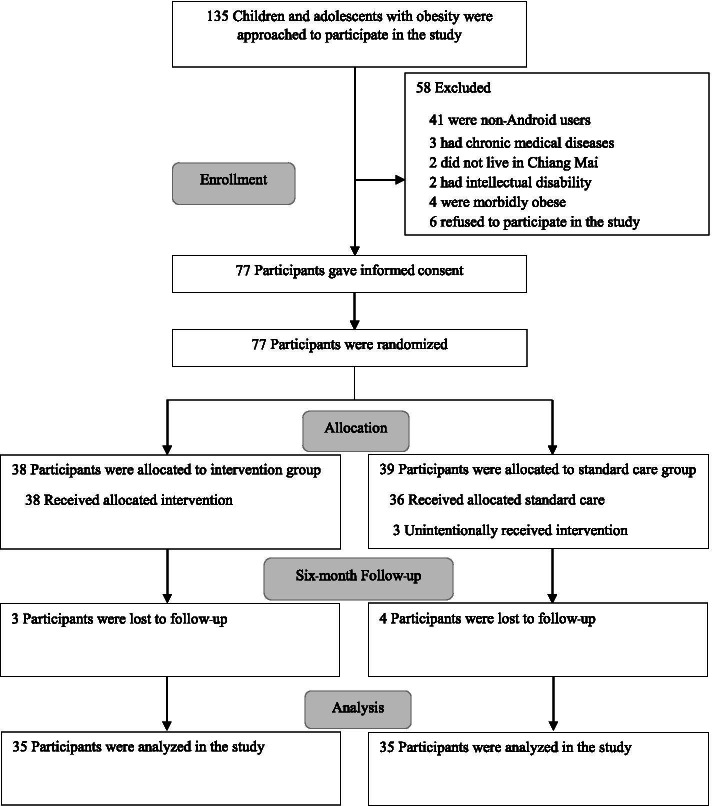
Study flow diagram

The participants were recruited from the hospital and school-based settings. For the hospital-based setting, the walk-in patients with minor illness were invited from the Pediatric Outpatient Department in Chiang Mai University Hospital. The school-based samples were students enrolled from a private school located in the same district as the hospital. One hundred thirty-five participants who met the inclusion criteria: age 10-15 years and obese (having BMI at or above the 95th percentile for children and adolescents of the same age and sex) were informed about the study by the research assistant. If children and their parents were interested in participating in the study, the research assistant would assess for eligibility based on the inclusion and exclusion criteria. The exclusion criteria consisted of 1) non-android device users; 2) having a chronic medical illness; 3) not living in Chiang Mai; 4) having an intellectual disability or requiring special educational needs; 5) not being ethnically Thai; and 6) being morbidly obese as they might usually require, at some point in time, additional treatments, intensive care, and hospitalization due to their comorbidities and complications during the study period [22], which could affect the primary and secondary outcomes measured in the study. After the exclusion, a total of 77 participants were asked to sign the informed consent and randomly allocated to the intervention or standard care group, as shown in the Consolidated Standards of Reporting Trials (CONSORT) flow diagram in Fig. 1.

### Intervention group

The participants in the intervention group were given the OBEST application, a newly developed mobile/tablet application as an additional tool for standard care, innovated by the research team. The strategic methodology of using this application was to improve adherence and promote motivation on reducing weight and changing eating behaviors to a healthier way. There were four features in this application: 1) Goal and Rewards, 2) Daily Dietary Record, 3) Tips and News, and 4) Messaging. Before using the application, the participants in this group were provided a two-hour workshop to learn the application’s instructions, the recommended diet, calories, portion sizes of food, and nutritional facts. Additional information shows the OBEST application in more detail (see Additional file 1).

### Standard care group

The participants received the standard treatment of obesity, followed by the Thai clinical practice guideline for childhood obesity treatment and prevention [23]. Participants from both the hospital and school-based settings were given the same standard of care by the healthcare providers.

### Outcome measurements

#### Anthropometric measurements

Bodyweight, height, BMI, BMI z-score, and waist circumference were measured three times in a row, and the mean of each measurement was calculated at the baseline and six-month follow-up. The bodyweight and height were measured using the NAGATA BW-110H digital weighing scale and the NAGATA BW-110H stadiometer. The non-stretch measuring tape measured the waist circumference. The participants would receive measurements at either hospital or school regarding the location of their first enrollment. The anthropometric parameters from the hospital and school settings were measured by the same nurse and the same standard measuring tools by each assessment time.

#### Healthy eating behaviors

The healthy eating behaviors were assessed using the healthy eating behavior questionnaire developed by the research team. This self-report questionnaire consisted of six eating habit items: 1) How many meals do you eat a day? 2) How often do you eat fruits and vegetables? 3) How often do you drink milk? 4) How often do you eat snacks? 5) How often do you drink sugar-sweetened beverages? and 6) How often do you eat fast food throughout a week? The three answers for each item were categorized into healthy and unhealthy habits. The answer ‘have three meals a day’ of item 1; ‘eat fruits and vegetables every day’ of item 2; ‘drink milk every day’ of item 3; ‘eat snacks less than three days a week’ of item 4; ‘drink sugar-sweetened beverages less than three days a week’ of item 5; and ‘eat fast food less than three days a week’ of item 6 were defined as having healthy eating habits. Apart from the above answers for each item referred to unhealthy eating habits.

#### Quality of life

The quality of life was assessed by the pediatric quality of life inventory™ version 4.0 generic core scales (PedsQL™). This is the universal tool for assessing the health-related quality of life in children [24]. The Thai version self-report questionnaire consists of 23 items that measure four-function domains: physical, emotional, social, and school. The psychosocial health summary scores were derived from combined mean scores of emotional, social, and school functioning domains. The mean scores of all functioning domains contribute to the total summary score. The higher mean scores indicate a better quality of life. The previous Thai research evaluated the psychometric properties of PedsQL 4.0 Generic Core Scales - Thai. Most of the domains in this tool had an acceptable level of internal consistency with Cronbach’s α of ≥0.65 [25].

### Sample size and power calculation

At least 24 children were needed in each group to detect a difference of 2.5 kg of bodyweight between intervention and control groups as presented in the previous study [26], with an alpha of 0.05 and power of 0.8. Due to the high attrition rate from weight management programs in the pediatric population, as seen in the previous study [27], we estimated to enroll the sample size of 77 participants (with anticipation for the drop-out rate of 50 - 60%).

### Statistical analysis

We used the SPSS program version 25.0 (IBM Corp, Armonk, NY) for statistical analysis. The independent samples *t*-test was used to test the statistical significance of changes of anthropometric measurements and PedsQL scores in six-month between-group differences. Odds ratios with 95% confidence interval were estimated to compare the number of participants engaging with healthy eating behaviors between the two groups. Intention-to-treat analysis was performed to investigate the difference in treatment effects on anthropometric measurements, healthy eating behaviors, and quality of life scores at six-month follow-up. The *p*-values of < 0.05 were considered statistically significant.

## Results

The baseline characteristics of the sample in the intervention and standard care groups are shown in Table 1. Most of the participants in the research study were boys. The mean age of all participants was 12.96 years.

**Table 1:** Baseline characteristics between groups

Characteristics	Intervention Group (***n*** = 35)	Standard Care Group (***n*** = 35)
	Mean (SD)	Mean (SD)
Male, *n* (%)	24 (69)	24 (69)
Age (years)	13.11 (1.99)	12.81 (1.79)
Weight (kg)	78.27 (20.56)	71.88 (15.43)
Height (cm)	160.94 (11.67)	157.74 (9.74)
BMI (kg/m^2^)	29.68 (4.09)	28.61 (3.74)
BMI z-score	2.72 (0.49)	2.61 (0.61)
Waist circumference (cm)	96.94 (12.17)	93.96 (10.09)
**Setting**		
Hospital-based sample, *n* (%)	19 (54)	18 (51)
School-based sample, *n* (%)	16 (46)	17 (49)
**Healthy eating habits**		
Having 3 meals/day, *n* (%)	31 (89)	25 (71)
Eating fruits and vegetables every day, *n* (%)	14 (40)	7 (20)
Milk consumption every day, *n* (%)	16 (46)	15 (43)
Less frequent consumption of snack (< 3 d/wk), *n* (%)	11 (31)	15 (43)
Less frequent consumption of sugar-sweetened beverages (< 3 d/wk), *n* (%)	19 (54)	19 (54)
Less frequent consumption of fast food (< 3 d/wk), *n* (%)	26 (74)	18 (51)
**PedsQL**		
Physical functioning	74.91 (16.63)	74.34 (14.54)
Emotional functioning	70.29 (16.49)	69.57 (14.11)
Social functioning	72.43 (19.98)	67.72 (20.63)
School functioning	72.14 (15.45)	68.42 (15.8)
Psychosocial health summary score	71.62 (12.59)	68.57 (12.29)
Total summary score	72.76 (12.02)	70.58 (11.98)

*kg* kilogram, *cm* centimeter, *m* meter, *BMI* body mass index, *d* day, *wk* week, *PedsQL* The Pediatric Quality of Life Inventory*™* version 4.0 Generic Core Scales, *SD* standard deviation

The anthropometric measurements are shown in Table 2. The magnitude of BMI change in the intervention group was greater than that in the standard care group but did not reach a significant level (− 0.62 [95% CI, − 1.03 to − 0.19] vs. -0.28 [95% CI, − 0.75 to 0.19]). A similar trend of BMI z-score changes was also found in the intervention group compared to the standard care group (− 0.2 [95% CI, − 0.27 to − 0.12) vs. -0.15 [95% CI, − 0.24 to − 0.06]). The remaining findings did not show significant differences between groups.

**Table 2:** Changes in growth parameters from baseline to 6-month follow-up between groups

Growth parameters	Intervention Group (***n*** = 35)	Standard Care Group (***n*** = 35)	***P-***value
	Mean (95% CI)	Mean (95% CI)	
Weight (kg)	1.55 (0.39, 2.73)	2.32 (1.06, 3.59)	0.42
BMI (kg/m^2^)	−0.62 (−1.03, −0.19)	−0.28 (− 0.75, 0.19)	0.33
BMI z-score	−0.2 (− 0.27, − 0.12)	−0.15 (− 0.24, − 0.06)	0.48
Waist circumference (cm)	0.17 (− 1.69, 1.82)	−0.23 (− 2.33, 1.66)	0.77

*kg* kilogram, *cm* centimeter, *m* meter*, BMI* body mass index, *CI* confidence interval

A secondary analysis was conducted to evaluate the post-treatment effect on growth parameters measured at 6-month follow-up between two groups using a generalized linear model approach. After adjusting for weight at baseline, the post-treatment weight of the intervention group was lower than the standard care group, but was not statistically significant (mean difference, − 0.53 [95% CI, − 2.43 to 1.37]), as shown in Supplementary Table 1 (See Additional file 2).

At a six-month follow-up, the study showed a higher percentage of participants engaging in healthy behaviors such as having three meals per day, eating fruits and vegetables every day, drinking milk every day, and less frequent consumption of fast food in the intervention group compared to the standard care group. Specifically, the number of participants engaging with less frequent consumption of fast food was significantly higher in the intervention group than in the standard care group (odds ratio, 4.5 [95% CI, 1.41 to 14.35]), as seen in Table 3.

**Table 3:** Number of participants engaging with healthy eating behaviors at 6-month follow-up between groups

Healthy eating behaviors	Intervention group (***n*** = 35)	Standard Care Group (***n*** = 35)	Odds ratio (95% CI)
Having 3 meals/day, *n* (%)	33 (94)	28 (80)	4.13 (0.79, 21.48)
Eating fruits and vegetables every day, *n* (%)	11 (31)	9 (26)	1.32 (0.47, 3.75)
Milk consumption every day, *n* (%)	15 (43)	11 (31)	1.64 (0.62, 4.35)
Less frequent consumption of snack (< 3 d/wk), *n* (%)	11 (31)	16 (46)	0.54 (0.21, 1.44)
Less frequent consumption of sugar-sweetened beverages (< 3 d/wk), *n* (%)	19 (54)	21 (60)	0.79 (0.31, 2.04)
Less frequent consumption of fast food (< 3 d/wk), *n* (%)	30 (85)	20 (57)	4.5 (1.41, 14.35)

*d* day, *wk* week, *CI* confidence interval

In Table 4, the participants from both groups reported the increased PedsQL scores in all domains over the 6 months of follow-up. There were no significant differences in pre-post treatment score changes between the two groups.

**Table 4:** Changes in quality of life scores from baseline to 6-month follow-up between groups

PedsQL	Intervention Group (***n*** = 35)	Standard Care Group (***n*** = 35)	***P-***value
	Mean (95% CI)	Mean (95% CI)	
Physical functioning	4.91 (− 2.55, 11.96)	1.91 (− 4.22, 7.43)	0.53
Emotional functioning	2.14 (−3.78, 7.94)	4.6 (−2.02, 10.03)	0.57
Social functioning	1.0 (−5.83, 7.56)	5.42 (0.51, 10.57)	0.29
School functioning	1.0 (−3.71, 5.14)	2.15 (−3.57, 8.09)	0.76
Psychosocial health summary score	1.38 (−3.2, 5.45)	4.06 (0.2, 8.06)	0.37
Total summary score	2.61 (−2.44, 6.81)	3.31 (−1.04, 7.28)	0.82

*PedsQL* The Pediatric Quality of Life Inventory*™* version 4.0 Generic Core Scales, *CI* confidence interval

In this study, 10% of participants lost to a six-month follow-up due to loss of motivation, relocation, and drop out with no reason given. The average compliance rate in using the OBEST application among participants in the intervention group was approximately 50%. This rate was measured by the frequency recording of daily food ingestion during the study period. The participants given the OBEST application would record their daily food types and portion sizes via the application. Then, an individual summary of daily food consumption was automatically sent to the physician’s computer software each day. Thus, the physician could monitor the calories intake and assess the frequency they recorded.

## Discussion

The present study could not find a significant difference in weight changing between the groups. However, it was found that the intervention group was significantly related to the increased trend of healthy eating behaviors – less frequent consumption of fast food as seen from the odds ratio. For the quality of life scores, both groups reported the increased PedsQL scores at six-month follow-up from baseline. There were no statistically significant changes in PedsQL scores over 6 months in between groups.

Since the mean weight of participants in the intervention group was higher at pre-treatment than the standard care group, we conducted a secondary analysis to estimate the post-treatment effect on reducing weight between the two groups by adjusting baseline weight. It was found that the post-treatment weight of the intervention group was lower than the standard care group, however the confidence intervals were wide including the null value. The possibility of diminishing treatment effectiveness might be explained by a lack of parental involvement in this study. This resulted in inconsistent findings with the previous research reporting significant weight loss of overweight adolescents and their parents when using the internet-based weight management program [28]. Another explanation might be treatment adherence. A prior study that assessed the short-term outcome (2 months) of this mobile application on anthropometric assessments showed the benefit of a decrease in BMI in the intervention group [29]. In contrast, this study did not see those effects at the six-month follow-up due to the decrease in adherence to treatment over time, in our opinion. The compliance rate of using the mobile application in the intervention group of this study was not as good as we expected. Nonetheless, this compliance rate might be a good representation of the real-world population using a mobile application for treating obesity.

Inadequate consumption of fruits and vegetables and excessive consumption of high-fat/high-sugar diets play a crucial role in childhood obesity [30]. This study showed that less than half of the participants consumed fruits and vegetables every day in intervention and standard care groups. The majority of children and adolescents with obesity did not drink milk every day. This could be due to the parents’ perception that milk was unnecessary as a child grew older. The possible problematic eating habits in children with obesity were eating snacks as often observed in more than half of the participants in both groups. We expected that children and adolescents with obesity would have more than three meals per day. Interestingly, our study found the participants primarily had up to three meals per day. However, having three regular meals was not always a good indicator of healthy eating behavior. Each meal might have a large portion size and contain a high-carbohydrate and high-fat diet. Besides, a minority of participants frequently consumed sugar-sweetened beverages and fast food, which was in contrast to previous studies [31, 32]. A possible explanation was the parent might not provide frequent fast food to their children due to its unaffordable price in our settings.

As expected, we found changing healthy eating behaviors, only in fast-food consumption, in the intervention group using the mobile application. The remaining healthy eating behaviors did not exhibit significant changes. The difficulty in changing behaviors and decreasing weight status could be explained by reducing treatment engagement over time [33]. Some behavior programs seem to be effective and feasible in the short-term treatment and follow-up course, as seen in the study of Chen et al. [34]. Therefore, healthy eating habit changes were usually not being observed in a longer period of time.

There were no statistically significant differences between pre-post treatment PedsQL scores between the two groups. The participants in both groups had higher scores at six-month follow-up compared with the baseline scores indicating that they had a better quality of life after receiving obesity treatment, which supports the finding in a previous study [35]. The increased physical functioning scores might be explained by participants perceived they were healthier, and the reduced BMI might, in turn, facilitate them engaging in more physical activities. Increased ratings in psychosocial health demonstrated in this study might indicate that participants would have a positive attitude towards their shape, which would promote their self-confidence, feeling of acceptance from peers, good relationships, and fewer problems of bullying [36].

This study has several strengths. This application was developed for children as distinct from other weight management applications mostly designed for adults. Also, all data input in the application were from reliable sources, unlike many mobile applications for weight management that lacked professional content input [37]. The application also focused on promoting self-awareness by providing a self-monitoring dietary record and building motivation by adding features in the application – the adjustable photo display feature, which showed how the participants looked when they were thin. The goals of using the application were to reduce weight and promote healthy eating behaviors. However, changing eating behaviors in children was challenging [38, 39]. This study showed a trend of enhancing some healthy eating behaviors when using application combined with standard treatment. Moreover, the dropout rate was relatively low in this study.

Nonetheless, there were some limitations to this study. Firstly, the eating behaviors questionnaire was not validated since it was developed by our research team used only for this study. However, a nutritionist reviewed the questionnaire as a relatively good substitute for the healthy eating habits evaluation. The questionnaire construction was based on the recommendation that is commonly offered in the general practice clinic. Secondly, in spite of randomization, participants in the intervention group still tended to be more obese at baseline than the standard care group. Although the difference of weight at baseline between the two groups did not reach a significant level, randomization failure by chance could occur in this study. It might explain the absence of the treatment effect. Due to the randomization process was not successful in producing equal distributions of confounding factors, the socioeconomic status variable, which was not collected in this study, could be a potential confounder in the study. The imbalance of initial weight between the two groups could also indicate a presence of unmeasured confounding factors, such as genetic effects, which were not evenly distributed among the groups. Thirdly, the possibility of type II error due to a relatively small sample size could be the one reason that no significant changes were found between intervention and control groups. Fourthly, having a relatively low compliance rate in using the application in the intervention group while still receiving the suggestion from the physician every follow-up visit in the standard care group might also dilute, to some extent, the effect of the application. Fifthly, there was a heterogeneity of samples from the hospital and school-based settings. To mitigate the effect of inhomogeneous samples, we performed stratified random sampling by using stratifying variables such as age and sex. Lastly, the OBEST application is in the Thai-only language and compatible with Android devices due to software development limitations. Thus, we implicitly excluded the children who did not understand the Thai written and spoken language and were not Android users.

## Conclusions

The current study did not find a significant effect of the OBEST application, as an adjunct tool to the standard treatment, on reducing weight in obese children and adolescents. However, this application might help to increase engaging in healthy eating behaviors. Studies with larger sample sizes are needed to support our findings. Future randomized clinical trials on this topic should consider the possibility of confounding due to chance after randomization.

## Supplementary Information

**Additional file 1.** Supplementary information on OBEST application.

**Additional file 2: Supplementary Table 1.** Growth parameters at 6-month follow-up between two groups.

## Data Availability

The datasets used and analyzed in this study are available from the corresponding author on reasonable request.

## Abbreviations

BMI: Body mass index
OBEST: The OBEST application
PedsQL: The Pediatric Quality of Life Inventory*™* version 4.0 Generic Core Scales
SD: Standard deviation
