# Supplementary material for: Effectiveness of mobile application on changing weight, healthy eating habits, and quality of life in children and adolescents with obesity: a randomized controlled trial
Source: BMC Pediatr. 2021 Nov 10;21:499. doi: 10.1186/s12887-021-02980-x (PMC8579514; doi:10.1186/s12887-021-02980-x)
Supplement: Supplementary file 1 — Additional file 1. Supplementary information on OBEST application. [file 12887_2021_2980_MOESM1_ESM.pdf]

# Additional file 1
# Supplementary information on OBEST application

**Description of Application**

OBEST is an application which is applicable on mobile phone and tablet devices. It is a newly developed application for use as an adjunctive tool to the standard care treatment of childhood obesity. This application is compatible with android-only devices. It is in the Thai language, free of charge, user-friendly, and suitable for children.

The underlying hypothetical strategy of constructing this application was to promote the patient's self-awareness by providing self-monitoring dietary intake records. This application also highlights the motivational concept because it can be a driving force for altering lifestyle behaviors [1]. The ultimate goals are assessed by achieving weight reduction and changing the old habits to healthier eating behaviors.

**Application Development**

The application developers comprised the developmental-behavioral pediatricians, a pediatric nutrition specialist, programmers, and technical computer officers. The pediatricians and pediatric nutrition specialist thoroughly reviewed the information constructing this application. The application's features and usage were approved by the Research Ethics Committee of Faculty of Medicine, Chiang Mai University.

**Application Features**

Initially, the physician enters the patient's data and chooses the 'weight reduction' program. The application will set the daily calorie intake and portion sizes of food adjusted for the patient's eating pattern.

OBEST has four features: 1) 'Goal and Rewards' is the feature that show the set goal of calorie intake and portion sizes of food recommended per day. The patient can collect gold stars as a daily reward by having no more than the set amount of calories throughout the day. It can remind the user to weigh him/herself every two weeks, and the user can review their progress over time. There is also a motivational feature, the adjustable photo display feature, which shows how they look when they are thin. 2) 'Daily Dietary Record' is the feature that the user can record their daily food ingestion, and it will summarize a total dietary calorie intake per day. This function is based on the Nutrifact [2], an accurate database providing the nutritional content of Thai foods established by the Research Institute for Health Sciences, Chiang Mai University. Moreover, the individual summary of food consumption was sent to the physician's computer software each day so the physician can track their patients. 3) 'Tips and News' are monthly updated information based on WHO recommendations to lose weight and to maintain healthy eating behavior. 4) 'Messaging' from the healthcare provider was sent similarly to the users every two weeks to remind and motivate them to keep up the intervention.

**Safety and Usage**

The personal data such as weight, height, age, and study code were passcode locked. Thus, no one could see this information except for the healthcare provider. The application was given to the intervention group participants for

the entire six months of intervention. We encouraged children and adolescents to record the data by themselves. Users recorded food intake either after each meal or once at the end of the day. The approximate time to record the data was 5 minutes. Due to the short time required and the ability to work in an offline mode, the parent’s concerns about their child’s inappropriate screen use were relatively low.

**Reliability and Validity**

**Reliability:** Each of the daily food records of five children was randomly paired on weekdays and weekends (i.e., for weekdays, Thursday on the 1$^{st}$ week paired with Thursday on 2$^{nd}$ week; for weekends, Sunday on the 1$^{st}$ week paired with Sunday on the 2$^{nd}$ week). We collected ten pairs of daily total calorie intakes consisting of five pairs of weekdays and five pairs of weekends. Daily total calorie intakes were adjusted to quartile. We did Cohen's kappa coefficient to test the agreement of dietary intakes. The kappa coefficient (k) was 0.72

**Validity:** We only test the face validity. We telephoned each parent of five children to check whether the contents of food records (types and portion sizes) that the children entered on weekdays and weekends were correct. The overall percentage of accuracy was approximately 70%. However, face validity is an informal and subjective assessment. The database of our application’s food choices was based on the National survey food consumption data of Thailand, National Bureau of Agricultural Commodity and Food Standards [3]. Therefore, the children would be familiar with the food choices in the application.

**References**

1. Sparud-Lundin C, Andersson K. Participation and motivation in obesity treatment – A qualitative study of teenagers’ and parents’ perceptions. Obes Control Ther. 2015;2(1):1-6. https://doi: 10.15226/2374-8354/2/1/00114
2. Research Institute for Health Sciences, Chiang Mai University. Nutrifacts: Nutritive values of foods. Chiang Mai. 2015. [in Thai]
3. National Bureau of Agricultural Commodity and Food Standards. Food consumption data of Thailand. 2016. [in Thai]
