# Supplementary material for: Effectiveness of mobile application on changing weight, healthy eating habits, and quality of life in children and adolescents with obesity: a randomized controlled trial
Source: BMC Pediatr. 2021 Nov 10;21:499. doi: 10.1186/s12887-021-02980-x (PMC8579514; doi:10.1186/s12887-021-02980-x)
Supplement: Supplementary file 2 — Additional file 2: Supplementary Table 1. Growth parameters at 6-month follow-up between two groups. [file 12887_2021_2980_MOESM2_ESM.pdf]

Additional file 2

Supplementary Table 1

Supplementary Table 1 Growth parameters at 6-month follow-up between two groups

**Growth parameters**	**Intervention Group (*n*=35) Mean (SD)**	**Standard Care Group (*n*=35) Mean (SD)**	**Crude Mean Difference (95% CI)**	**Adjusted Mean Difference* (95% CI)**	***p*-value***
Weight (kg)	79.82 (20.07)	74.2 (15.46)	5.62 (-2.93, 14.16)	-0.53 (-2.43, 1.37)	0.58
BMI (kg/m^2)	29.07 (4.2)	28.32 (4.06)	0.74 (-1.23, 2.71)	-0.42 (-1.62, 0.78)	0.48
BMI z-score	2.52 (0.56)	2.45 (0.67)	0.06 (-0.23, 0.36)	-0.04 (-0.3, 0.23)	0.77
Waist circumference (cm)	97.11 (11.5)	93.73 (9.56)	3.39 (-1.66, 8.43)	0.23 (-2.5,2.97)	0.87

*Adjusted for weight at baseline
